# Impact of miR-21, miR-126 and miR-221 as Prognostic Factors of Clear Cell Renal Cell Carcinoma with Tumor Thrombus of the Inferior Vena Cava

**DOI:** 10.1371/journal.pone.0109877

**Published:** 2014-10-03

**Authors:** Daniel Claudius Vergho, Susanne Kneitz, Charis Kalogirou, Maximilian Burger, Markus Krebs, Andreas Rosenwald, Martin Spahn, Andreas Löser, Arkadius Kocot, Hubertus Riedmiller, Burkhard Kneitz

**Affiliations:** 1 Department of Urology and Pediatric Urology, Julius Maximilians University Medical Center, Würzburg, Germany; 2 Physiological Chemistry I, Biocenter, University of Würzburg, Würzburg, Germany; 3 Department of Urology, University of Regensburg, Regensburg, Germany; 4 Department of Pathology, Comprehensive Cancer Center Mainfranken (CCCM), University of Würzburg, Würzburg, Germany; 5 Department of Urology, University Hospital Bern, Bern, Switzerland; University Hospital Carl Gustav Carus Dresden, Germany

## Abstract

Clear cell renal cell carcinoma (ccRCC) characterized by a tumor thrombus (TT) extending into the inferior vena cava (IVC) generally indicates poor prognosis. Nevertheless, the risk for tumor recurrence after nephrectomy and thrombectomy varies. An applicable and accurate prediction system to select ccRCC patients with TT of the IVC (ccRCC/TT) at high risk after nephrectomy is urgently needed, but has not been established up to now. To our knowledge, a possible role of microRNAs (miRs) for the development of ccRCC/TT or their impact as prognostic markers in ccRCC/TT has not been explored yet. Therefore, we analyzed the expression of the previously described onco-miRs miR-200c, miR-210, miR-126, miR-221, let-7b, miR-21, miR-143 and miR-141 in a study collective of 74 ccRCC patients. Using the expression profiles of these eight miRs we developed classification systems that accurately differentiate ccRCC from non-cancerous renal tissue and ccRCC/TT from tumors without TT. In the subgroup of 37 ccRCC/TT cases we found that miR-21, miR-126, and miR-221 predicted cancer related death (CRD) accurately and independently from other clinico-pathological features. Furthermore, a combined risk score based on the expression of miR-21, miR-126 and miR-221 was developed and showed high sensitivity and specificity to predict cancer specific survival (CSS) in ccRCC/TT. Using the combined risk score we were able to classify ccRCC/TT patients correctly into high and low risk cases. The risk stratification by the combined risk score (CRS) will benefit from further cohort validation and might have potential for clinical application as a molecular prediction system to identify high- risk ccRCC/TT patients.

## Introduction

ccRCC represents 2–3% of all solid neoplasms with a worldwide annual increase in incidence of about 2% [1]. About 5-10% of ccRCCs extend into the renal vein or the IVC [2]. When this occurs without evidence of lymph node involvement or distant metastasis, surgery offers the only potential cure [3], whereas patients who present with metastatic disease have a poor prognosis with a 5-year survival rate of less than 20% [4]. The 5-year survival rates for patients with ccRCC/TT without evidence of nodal or distant metastasis treated with nephrectomy and tumor thrombectomy is 46% −65% [4], [5], [6], [7]. Several studies have evaluated the prognostic value of clinico-pathological features like performance status, presence of metastasis, sarcomatoid features, concomitant perinephritic fat invasion, tumor grade, level of TT and histological subtype in ccRCC patients with venous involvement [6], [7], [8].

Nevertheless, the impact of molecular markers in this setting has been insufficiently studied up to now.

While a couple of biological markers have been tested and validated in the attempt to improve risk stratification for ccRCC patients [9], only limited data is available concerning ccRCC/TT patients. Recently, Laird et al. reported on differential expression of prognostic proteomic markers in primary tumor, venous TT and metastatic ccRCC tissue. Ki67, p53, VEGF1 (vascular endothelial growth factor 1), SLUG and SNAIL were significantly higher expressed in metastases compared with primary tumor and TT, but no difference between primary tumor and TT was seen [10].

Establishment of adjuvant therapy concepts for ccRCC/TT patients after radical surgical treatment have been hindered by lacking reliability of prediction of outcome by both clinical and molecular parameters to this date [9]. Because of that, the identification of novel markers is urgently needed if harbouring personalized therapy and follow-up.

One current approach for molecular tumor characterization is miR expression profiling [11]. MiRs are small noncoding RNA strands that posttranscriptionally regulate gene expression and appear to be modulators of urologic cancers [12]. Specific miR profiles have been observed previously in ccRCC: we could show recently that a combined risk score (CRS) of miR-21 and miR-126 accurately predicts survival in ccRCC cases [13].

Based on the existing literature we selected a panel of eight miRs (miR 200c, miR-210, miR-126, miR-221, let-7b, miR-21, miR-143, and miR-141) that were shown to be dysregulated in ccRCC to analyse their expression in a study cohort containing ccRCC/TT and ccRCC without TT (ccRCC/woTT) cases.

Here, we assessed expression of eight oncogenic miRs to determine an expression profile which allowed us to distinguish between ccRCC/TT patients and ccRCC patients not having vascular invasion. To evaluate the potential role of miRs as prognostic molecular markers in ccRCC/TT patients we finally correlated the expression of selected miRs with clinico-pathological features and survival aiming towards a possible clinical use as molecular markers.

## Materials and Methods

### Ethics statement

This study was approved by the local human research ethics committee of the medical faculty of the University of Wuerzburg, Germany (no. 136/08) and was conducted according to the standards set by the declaration of Helsinki; all patients provided written informed consent.

### Patients and tissue sample

We collected paraffin embedded samples of 74 primary tumors of ccRCC patients. After excluding other histological histological subtypes (7 papillary, 3 sarcomatoid) we used samples of 37 primary tumors of ccRCC/TT patients who consecutively underwent radical surgery at the Department of Urology and Pediatric Urology of the Julius-Maximilians-University Medical Center Würzburg between 1997–2010.

To evaluate the role of the selected miRs in development of venous involvement, a comparison collective of consecutive ccRCC cases without venous invasion (n = 37) was used. All ccRCC samples were paraffin-embedded and areas with >90% cancerous tissue were selected; likewise samples of histologically benign renal tissue were reviewed by one experienced uropathologist (AR) and used non-cancerous renal tissue as controls. ccRCC specimens were staged and graded according to the TNM classification (2010 TNM classification of malignant tumors (UICC, 7^th^ edition) by a uropathologist (AR). The level of tumor thrombus was classified according to the Mayo classification [14] Clinical and pathological characteristics including follow up are summarized in Table 1.

**Table 1:** Patient characteristics (n = 74).

Characteristics	Entire ccRCCgroup (n = 74)	ccRCC patientswithout TT (n = 37)	ccRCC patientswith TT (n = 37)
Median Follow up	45.6 months	45.7 months	41.4 months
Median Age	66.8 years	68.0 years	65.6 years
Sex			
Female	26 (35.1%)	17 (45.9%)	9 (24.3%)
Male	48 (64.9%)	20 (54.1%)	28 (75.7%)
Tumor Grade			
G1	4 (5.4%)	4 (11%)	0
G2	52 (70.2)	30 (81%)	22 (59,5%)
G3	16 (21.6%)	3 (8%)	13 (35%)
G4	2 (2.7%)	0	2 (5.5%)
T stage			
T1a	12 (16.2%)	12 (32.4%)	0
T1b	12 (16.2%)	12 (32.4%)	0
T2a/b	8 (10.8%)	8 (21.6%)	0
T3a	5 (6.8%)	5 (13.5%)	0
T3b	30 (40.5%)	0	30 (81.1%)
T3c	7 (9.6%)	0	7 (18.9%)
T4	0	0	0
N stage			
N0	70 (94.6%)	37 (100%)	33 (89.2%)
N1	1 (1.4%)	0	1 (2.7%)
N2	3 (4.1%)	0	3 (8.1%)
Metastasis at time of surgery			
Yes	18 (24.3%)	2 (5.4%)	16 (43.2%)
No	56 (75.7%)	35 (94.6%)	21 (56.8%)
Level of Tumor Thrombus (Mayo-classification)			
no TT	37 (50%)	37 (100%)	0
Level I	5 (6.8%)	0	5 (13.5%)
Level II	10 (13.5%)	0	10 (27%)
Level III	15 (20.3%)	0	15 (40.5%)
Level IV	7 (9.5%)	0	7 (18.9%)
Infiltration of perinephritic tissue			
yes	21 (28.4%)	5 (13.5%)	16 (43.2%)
no	53 (71.6%)	32 (86.5%)	21 (56.8%)
Clinical failure			
Yes	28 (37.8%)	3 (8.1%)	25 (67.6%)
No	46 (62.2%)	34 (91.9%)	12 (32.4%)
Cancer related death			
Yes	21 (28.4%)	1 (2.7%)	20 (54.1%)
No	53 (71.6%)	36 (97.3%)	17 (45.9%)

Preoperatively, all patients underwent routine blood test, ultrasound, chest x-ray (or computed tomography (CT)), abdominal CT and/or abdominal magnetic resonance imaging (MRI) and/or bone scintigraphy. Long-term follow-up data were collected during check-up visits, review of patient records and additional telephone interviews with the urologists of the patients.

### RNA Extraction and Reverse Transcription

Total RNA extraction from paraffin-embedded samples was performed using the Recover all Total Nucleic Acid Isolation Kit and the Total RNA Extraction Kit, respectively (Ambion and miRNeasy Mini Kit, Qiagen). RNA concentration and A260/280 ratio were analysed with a Nano Drop ND-100 spectrometer (NanoDrop Technologies, Wilmington) and RIN (RNA Integrity Numbers) calculated with a Bioanalyzer. RNA samples showing RIN<6.0 were excluded from further analysis. The resulting miR was retained for quantitative Real Time PCR (qRT-PCR). Specific cDNA was synthesized from total RNA with stem-loop reverse transcription primers according to the TaqMan miRassay protocol (PE Applied Biosystems).

### qRT-PCR

MiR expression in tissue samples was quantified with TaqManH miRassay kits and the BioRad OPTICON 2, following the manufacturer’s instructions (BioRad). Primers for all miRs were obtained from Applied Biosystems. Cycling conditions were chosen according to manufacturer’s protocols. All reactions were performed in triplicates and samples showing SD>0.5 were excluded. Relative expression values of miRs were normalized to small nuclear RNA (RNU6b) previously described as reference gene. ΔC_t_ for tumor samples and adjacent normal tissue of all miRs were calculated by the comparative C_t_ method. All samples characterized by expression levels of RNU6B>30 C_t_ were excluded from further analysis.

### Statistic, computional analysis and combined risk score calculation

Thresholds for dichotomizing relative expressions of miRs were determined by receiver operating characteristic (ROC) curves (R package pROC [15]), based on CSS. Impact of clinic-pathological parameters and various miRs on CSS was assessed by uni- and multivariate COX regression analysis (R-package survival, [16]). The best fitting COX model was selected by measuring the relative goodness-of-fit with the Akaike information criterion (AIC), which selected a combination of miR-21 miR-126 and miR-221 as the best predictor. Calculation of a CRS of miR-21 miR-126 and miR-221 was implemented as proposed by Lossos et al. [17]. In brief, a factor derived from the z-score, resulting from the COX model, was determined for all three miRs. The relative expression (ΔC_t_) of the different miRs were multiplied by these factors using the formula (4.592× ΔC_t_ miR-21)+(−3.892× ΔC_t_ miR-126)+(−1.938× ΔC_t_ miR-221). A negative factor indicates that higher expression correlates with longer survival, whereas a positive factor correlates with shorter survival. A cut-off for the risk score was again determined by ROC. Differences in mean between miR-expression and clinical parameters were analysed by Student's t-test or ANOVA, respectively.

## Results

### Developing a classification model for discrimination of ccRCC from normal kidney tissue based on miR expression profiles

To analyze the expression of the selected oncomiRs (miR-200c, miR-210, miR-126, miR-221, let-7b, miR-21, miR-143 and miR-141) we used the entire collective (n = 106) including 37 cases of ccRCC/TT patients and 37 cases without venous involvement (ccRCC/woTT) as well as 32 cases of non-cancerous renal tissue. We previously excluded all renal cell cancer (RCC) histo-pathological subtypes others than ccRCC from our study collective, as it was shown that different RCC subtypes are characterized by distinct miR expression profiles [18], [19]. Mean expression of the selected eight miRs in the ccRCC samples were calculated using normalized qRT-PCR data and compared to the mean expression in normal kidney tissue (n = 32) (Fig. S1). We observed significant upregulation of miR-21 and miR-210 in ccRCC samples, while miR-141, miR-200c and miR-126 were found to be downregulated in ccRCC cases. MiR-143, miR-221 and let-7b showed no significant differential expression (p>0.05).

To prove whether the expression profiles of the selected miRs correctly discriminate ccRCC tissue from non-cancerous tissue, we developed predictive rules using logistic regression. Therefore, we randomly divided the study collective (n = 106) into a learning data set of 58 samples containing 42 ccRCC samples and 16 samples of normal kidney tissue and a test data set containing the remaining 48 samples (32 ccRCC cases and 16 samples from non-cancerous kidney tissue). A combination of five miRs (miR-21, miR-143, miR-200c, miR-210 and miR-126) was determined by the AIC to accurately discriminate between normal kidney and ccRCC samples (ccRCC/TT and ccRCC/woTT together) in the learning data set. The area under the curve characteristics predicted 100% sensitivity and specificity (AUC = 1.00; p<0.001, Tab. 2). We then applied the classification model to the test cohort containing the remaining 32 tumor samples and 16 normal tissues. Using our model for differentiation the accuracy to discriminate between tumor and normal tissue was 100% in the testing cohort as indicated in table 2. Though, we concluded that the combination of these five miRs accurately differentiates ccRCC cases from normal kidney tissue in our study collective.

**Table 2:** Classification properties of miRNA for ccRCC/TT.

		Learning set				
Discrimination	n	miRNAs	AUC	95% CI	P-value	Correct classification
RCC vs.normal renal tissue	58 (42 tumor vs.16 normal)	miR-21, miR-143, miR-200c,miR-210, miR-126	1.00	100–100	<0.0001	58/58 (100%)
RCC/TT vs.RCC/woTT	37 (19 RCC/TT vs.18 ccRCC/woTT)	let-7b, miR-21, miR-221	0.89	77.6-100	<0.001	19/19 (100%)
		**Test set**				
**Discrimination**	**n**	**miRNAs**	**AUC**	**95% CI**	**P-value**	**Correct classification**
RCC vs.normal renal tissue	48 (tumor 32 vs.normal 16)	miR-21, miR-143, miR-200c,miR-210, miR-126	1.00	100–100	<0.0001	48/48 (100%)
RCC/TT vs.RCC/woTT	37 (18 RCC/TT vs.19 ccRCC/woTT)	let-7b, miR-21, miR-221	0.82	68.15–96.18	0.002	17/18 (94%)

Abbreviations: RCC : renal cell carcinoma; TT : tumor thrombus; woTT : without tumor thrombus; AUC: area under receiver characteristic curve; normal: non-cancerous renal tissue.

### Identification of miRs differentially expressed in ccRCC/TT

**Figure 1:**
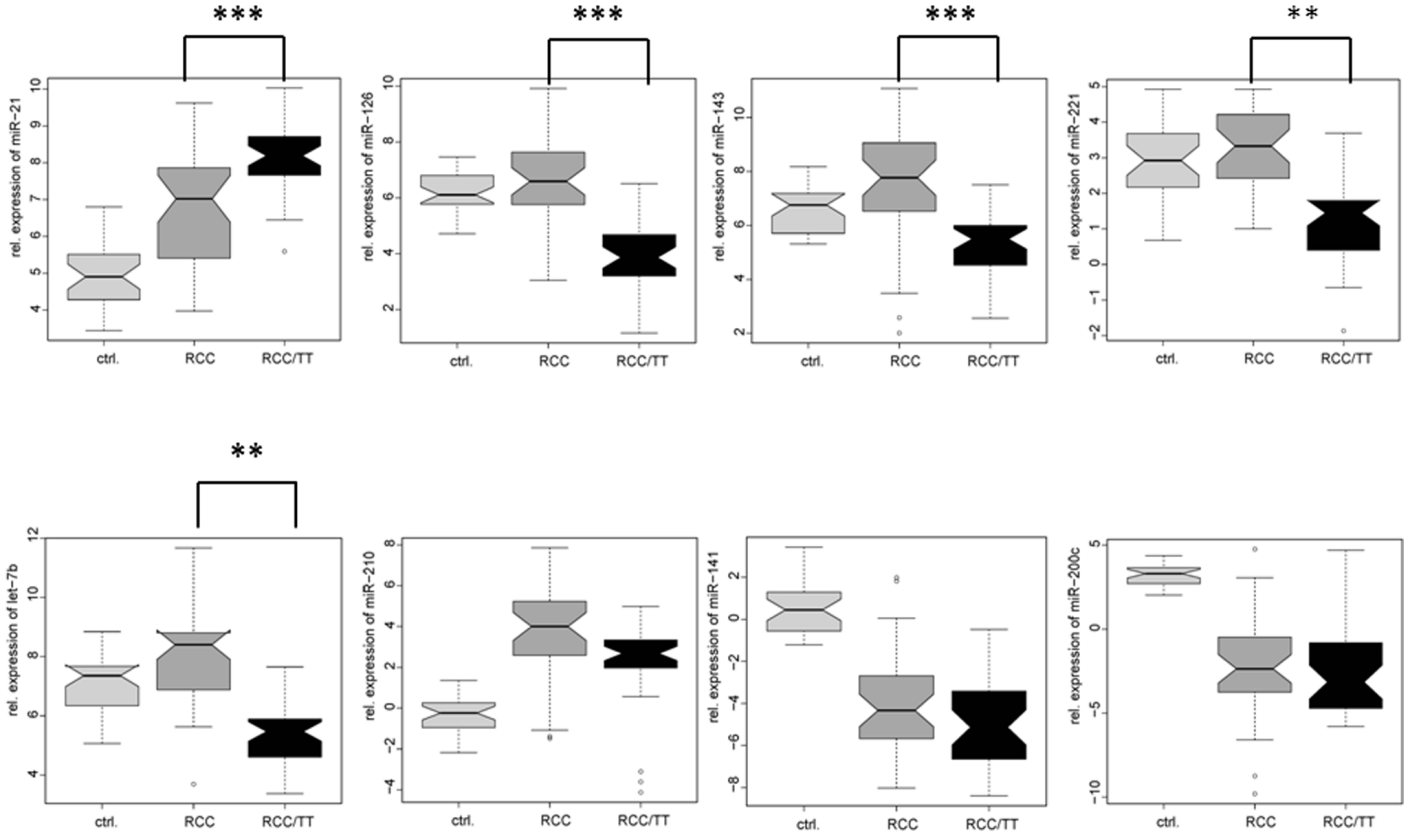
Expression of miR-21, miR-126, miR-143, miR-221, let-7b, miR-210, miR-141, and miR-200c in ccRCC/TT. Relative expression (ΔC_t_ levels) of indicated miRs measured by qRT-PCR and normalized against RNU6B are shown as Box and Whisker-Plot. Expression of different miRs in ccRCC/woTT (RCC, dark grey plots; n = 37) are compared with ccRCC/TT (black plots, n = 37). Expression in non-cancerous renal tissue (ctrl.; light grey plots, n = 31) was shown as control. * P<0.05; ** P<0.01; *** P<0.001, ANOVA.

To identify miRs, which are specifically dysregulated in ccRCC/TT cases, we compared the mean expression of the selected miRs in these cases (n = 37) to non-cancerous renal tissue. As shown in Fig. 1 we observed differential expression of all eight miRs analyzed in ccRCC/TT when compared to non-cancerous kidney tissue.

**Figure 2:**
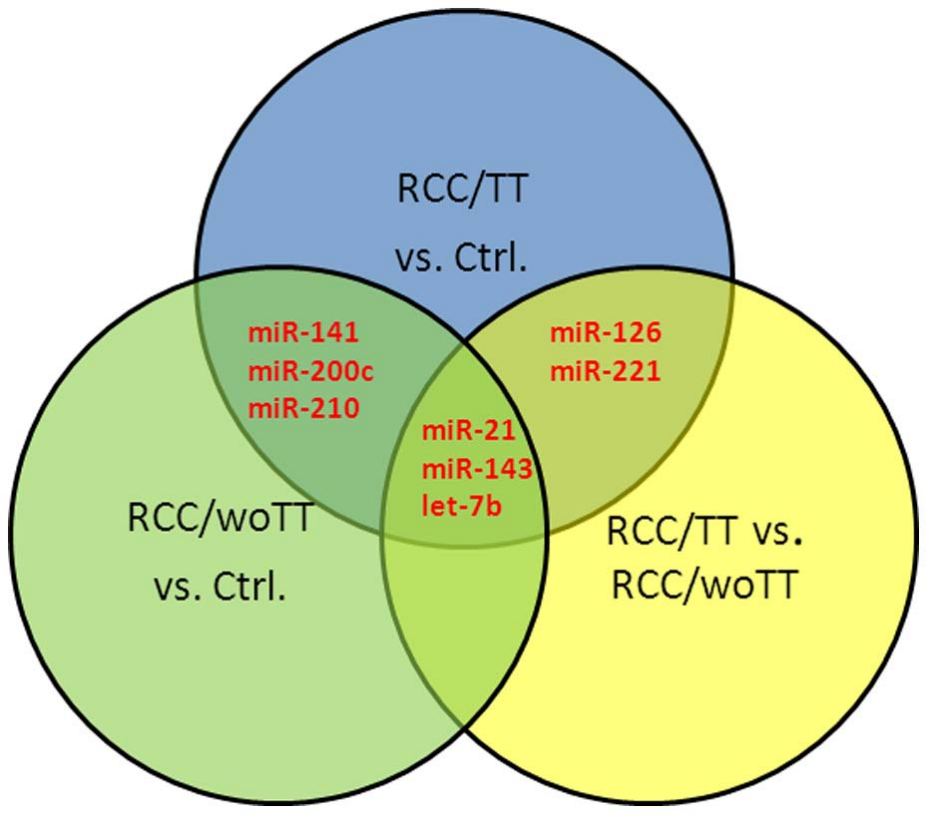
Venn diagram showing relationships between miRs that were differentially expressed in ccRCC/TT, ccRCC/woTT and non-cancerous renal tissue (ctrl). Circles include up- or down- regulated miRs for each pairwise comparison. Common miRs between different comparisons are shown in the intersections.

Next, we divided our study group into ccRCC/TT and ccRCC/woTT cases (table 1). Comparing both groups we found significant up-regulation of miR-21 and down-regulation of let-7b, miR-126, miR-221, and miR-143 in ccRCC/TT cases (Fig. 1). The mean expression of miR-141, miR-200c, and miR-210 showed no differential expression between both subgroups, but was significantly regulated, if each group was compared separately to non-cancerous kidney tissue. In contrast, miR-126 and miR-221 were down-regulated specifically in ccRCC/TT, but not in ccRCC/woTT as compared to the controls. Fig. 2 summarizes the differential expression of all miRs analyzed comparing ccRCC/TT cases, ccRCC/woTT cases and normal renal kidney using a Venn diagram. In addition to the specific down-regulation of miR-126 and miR-221 in ccRCC/TT cases we found a very robust progressive up-regulation of miR-21 in ccRCC/TT suggesting that miR-21, miR-126 and miR-221 might be specifically involved in the development of ccRCC/TT.

### Developing a classification model for discrimination of ccRCC/TT samples from ccRCC/woTT samples

Next, we evaluated whether the expression of these eight miRs could accurately discriminate ccRCC/TT samples (n = 37) from samples without invasion into the venous system (n = 37). Therefore, we developed a new classification model by logistic regression using a learning data set containing 37 ccRCC samples (19 ccRCC/TT and 18 ccRCC/woTT cases), which was randomly selected from the entire set of our study collective. By linear regression analysis, a combination of miR-21, miR-221 and let-7b was selected. All three together contributed essentially to the predicting model. Using the expression data of these three miRs, our model accurately determined ccRCC/TT samples with high sensitivity and specificity (AUC = 1.00; P<0.001, Tab. 2) in the learning cohort. To validate the discriminative properties of this classification model, we used an independent testing cohort containing the remaining 37 primary ccRCC cases (18 ccRCC/TT and 19 ccRCC/woTT). Using the defined parameters, this model correctly classified 17 of 18 (94%) ccRCC/TT cases. To further determine the properties of the model, we performed ROC analysis. Using the determined logistic regression calculations the AUC was 0.82 indicating a robust prediction of ccRCC/TT cases by the combined expression profile of miR-21, miR-221 and let-7b (see table 2).

### Association of miR expression with ccRCC/TT aggressiveness

**Figure 3:**
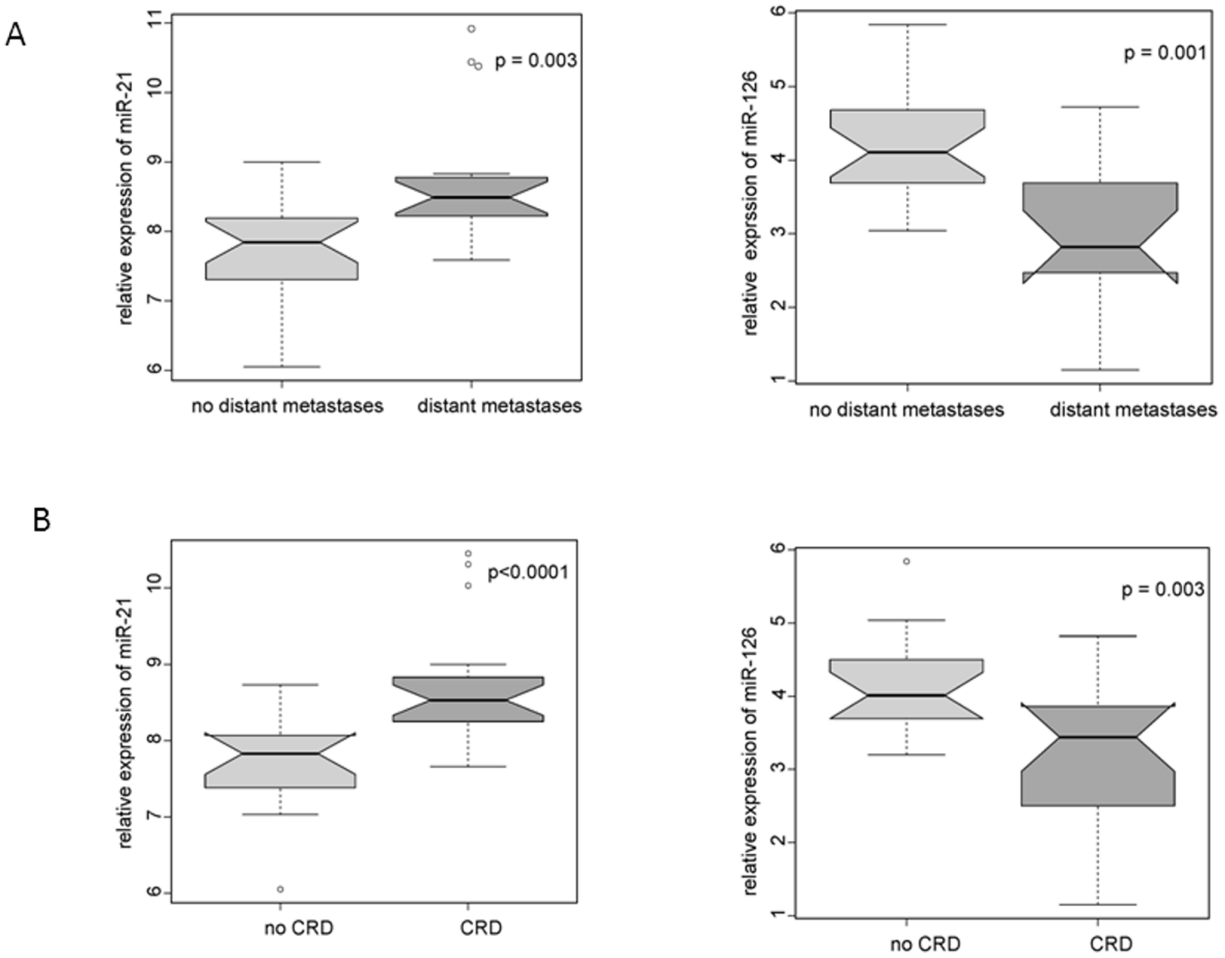
MiR-21 and miR-126 expression is associated to positive LN metastases and survival. Relative expression (ΔC_t_ levels) of miR-21 and miR-126 were analysed by qRT-PCR in ccRCC/TT samples and normalized using RNU6B. ccRCC/TT cases (n = 37) were divided into risk groups by positive distant metastases (A) or cancer specific death throughout follow up (B). Significant changes in median expression for miR-21 and miR-126 in between subgroups were calculated by unpaired student’s t-test and indicated in the Box and Whiskers plots.

To evaluate the possible impact of specific miRs as potential outcome predictor within the ccRCC/TT collective, we associated the expression of all eight miRs with positive distant metastasis at time of surgery (16 of 37 ccRCC/TT cases; table 1) and to CSS throughout follow up (20 of 37 ccRCC/TT cases; table 1) using the ccRCC/TT study collective (n = 37). The median follow up of the ccRCC/TT collective was 41.4 months with an actuarial 5-yr cancer specific survival estimate of 43% (Fig. S2). As shown in Fig. 3 and table 3, we observed significant up-regulation of miR-21 and down-regulation of miR-126 in ccRCC cases with metastasized disease at time of surgery or with CSS during follow up, while all other miRs did not show significant association to metastasis or survival (table 3).

**Table 3:** Differential expression of indicated miRs in risk groups divided by positive distant metastases at surgery (16 out of 37) and by cancer related death (CRD) (20 out of 37) throughout the follow up.

	distant metastases(mean expression ΔC_t_)				CRD(mean expression ΔC_t_)			
miRNA	yes	no	log2 ratio	p value	yes	no	log2 ratio	p value
let-7b	5.33	5.44	−0.11	0.96	5.54	5.26	0.28	0.79
miR-21	8.77	7.67	1.09	0.003 **	8.59	7.78	0.82	<0.0001 ***
miR-143	5.19	5.69	−0.50	0.66	5.50	5.24	0.25	0.80
miR-141	−5.15	−4.19	−0.95	0.51	−4.62	−4.96	0.34	0.80
miR-200c	−2.62	−2.11	−0.50	0.85	−2.50	−2.04	−0.46	0.38
miR-210	2.15	2.57	−0.43	0.68	2.91	1.68	1.23	0.16
miR-126	3.02	4.20	−1.17	0.001 **	3.21	4.13	−0.93	0.003*
miR-221	1.29	1.24	0.05	0.55	1.35	1.58	−0.23	0.22

p<0.05 *; p<0.001**; p<0.0001*** unpaired students t test;

### Correlating miR signatures with cancer specific survival in ccRCC/TT

**Figure 4:**
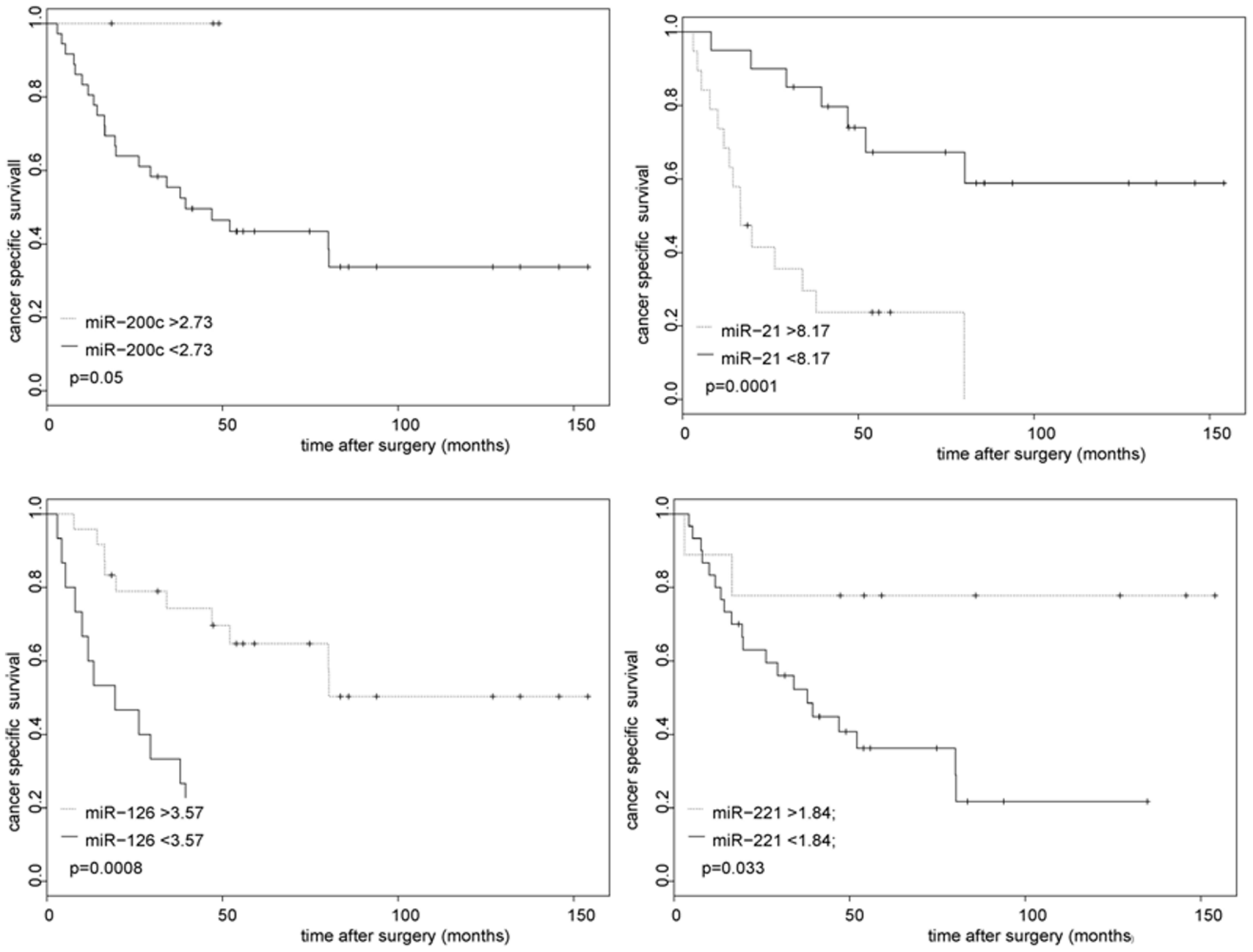
Kaplan Meier survival analysis for CSS in ccRCC/TT (n = 37) patients stratified by the dichotomized expression of miR- 200c, miR-21, mir-126 and miR-221. Risk scores (thresholds) for the miRs were determined by receiver operating characteristics (ROC) curves and indicated in the plots. The ccRCC/TT study cohort (n = 37) was stratified by the CRS of miR-21, miR-126 and miR-221. p values resulting from log rank tests are shown in the plots.

**Figure 5:**
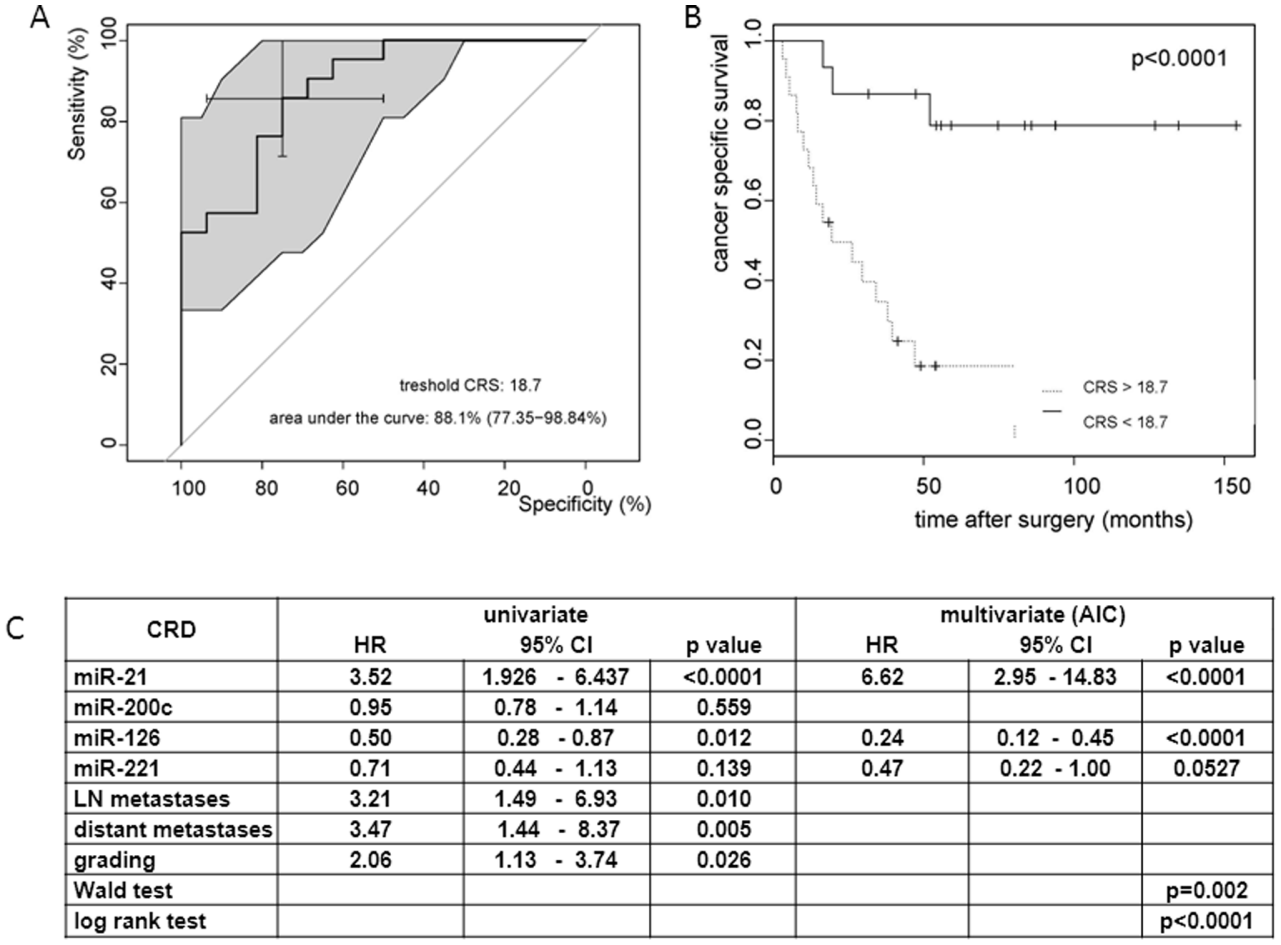
Kaplan Meier survival analysis and receiver operating characteristics (ROC) curve for CSS in ccRCC/TT patients (n = 37) stratified by the CRS for miR-21, miR-126 and miR-221. CRS were calculated as described in the material and methods part. A) ROC curve; the cross indicates the calculated cutoff score for the CRS resulting in the highest sensitivity and specificity The selected cut off score is indicated in graph B) The ccRCC/TT study cohort (n = 37) was stratified by the calculated risk score using Kaplan Meier analysis. Kaplan Meier curves with log rank test and risk stratification by CRS are shown. C) Uni- and multivariate Cox regression analysis for cancer related death in the ccRCC/TT collective determined by relative goodness of fit with AIC including selected miRs and clinico-pathological factors as variable.

ccRCC/TT, as expected, features markedly different biological behavior. While around 43% of all cases from the ccRCC/TT study group seem to be cured by aggressive surgery throughout 5-yr follow-up time, around 57% of these cancers recurred early and ultimately metastasized (Fig. S2). To determine which standard clinico-pathological risk factors should be integrated into a predictive algorithm to stratify patients at high risk for tumor recurrence, we performed Cox regression analysis using several risk factors including sex, age, tumor thrombus level, tumor grade, tumor size, perinephric fat infiltration, positive LN metastasis or positive distant metastasis at time of surgery. Only tumor grade, positive LN metastasis, and positive distant metastasis were univariately predicting CSS in the study group significantly and were therefore chosen for further analysis. To determine which of the eight miRs might be useful in predicting CSS thus being incorporated into a predictive algorithm consequently, we calculated Kaplan Meier plots and Cox regression analysis for all eight miRs using miR expression data (Fig. 4 and table 4). Expression differences in miR-21 and miR-126 significantly influenced CSS on Kaplan Meier estimates and univariate Cox regression analysis, while miR-200c and miR-221 were moderately significant to predict CSS in Kaplan Meier analysis. Therefore, we decided to combine miR-21, miR-126, miR-200c and miR-221 with the clinico-pathological factors tumor grade, LN metastases and distant metastases to develop a prediction model for CSS in ccRCC/TT patients. The potential of this model to predict CSS was evaluated by Cox regression analysis and Kaplan Meier estimates. To avoid overfitting of the model, we performed stepwise regression analysis using AIC resulting in a best model predicting CSS. This model used miR-21, miR-126 and miR-221 as multivariate significant factors indicating that these factors were independent predictors of CSS in our study collective (Fig. 5C). All clinico-pathological factors were not chosen by the AIC based model suggesting they were not essential for the predicting model. Using a previously described risk score model [17], we determined and calculated a combined risk score (CRS) for CSS based on the expression data of miR-21, miR-126 and miR-221 (used formula: (4.592× ΔCt miR-21)+(−3.892× ΔCt miR-126)+(−1.938× ΔCt miR-221)). The calculated CRS cut of level (high risk: CRS>18.7, low risk: CRS<18.7) divided the ccRCC/TT study cohort of 37 cases in high risk (n = 22) and low risk (n = 15) patients. Kaplan Meier plots and log rank tests showed stratification by the model for predicting patient survival (log rank p<0.001). Out of the 20 CRD cases, the risk score correctly identified 18 cases as high risk patients (90% specificity) and out of 16 cases with long term survival and without CRD throughout follow-up 14 cases were correctly classified as low risk patients (87% specificity). The predicted two and 5 year cancer free survival in patients divided by the CRS were 49% and 18% in the high risk group and 84% and 78% in the low risk group respectively, indicating that the CRS robustly predicts survival and CRD in ccRCC/TT patients. Additionally we observed that all 37 ccRCC samples without TT were correctly classified by the determined CRS into 36 cases at low risk and one case at high risk (data not shown). To test the performance of the predictive roles for classification of RCC with TT we finally analyzed the expression of miR-21, miR-126 and miR-221 in eight independent RCC/TT cases and calculated the CRS for each cancer patient. As shown in table 5 the CRS correctly classified 6 out of 7 high risk patients and one out of one patient at low risk for cancer progression indicating that 7 out of 8 patients (87%) were correctly classified by the CRS.

**Table 4:** Univariate Cox regression of ccRCC/TT patients for indicated miRs and clinico-pathological factors.

CRD	univariate		
	HR	95% CI	p value (Likelihood ratio test)
**let-7b**	1.20	0.7003 2.055	**p = 0.507**
**miR-21**	3.52	1.926 6.437	p<0.0001***
**miR-143**	0.97	0.667 1.419	**p = 0.885**
**miR-141**	1.02	0.8064 1.286	**p = 0.880**
**miR-200c**	0.95	0.7815 1.145	**p = 0.558**
**miR-210**	1.14	0.9068 1.435	**p = 0.231**
**miR-126**	0.50	0.2821 0.8686	p = 0.012 *
**miR-221**	0.71	0.4449 1.135	**p = 0.139**
**LN metastases**	3.21	1.491 6.929	p = 0.010 *
**distant metastases**	3.47	1.438 8.367	p = 0.005 *
**perinephric fat invasion**	1.44	0.6001 3.431	**p = 0.411**
**grading**	2.06	1.129 3.743	p = 0.026 *
**tumor size**	0.97	0.8431 1.115	**p = 0.660**
**tumor thrombus level**	1.10	0.7099 1.689	**p = 0.680**
**age**	1.04	0.9835 1.104	**p = 0.157**
**gender**	0.97	0.3782 2.48	**p = 0.946**

p<0.05 *; p<0.001**; p<0.0001***.

**Table 5:** Risk stratification for cancer related death (CRD) of eight RCC/TT patients by the combined risk score (CRS).

patient ID	CRD whilefollow up	time to CRD or lastfollow up (months)	miR-21 (ΔCt)	miR-126 (ΔCt)	miR-221 (ΔCt)	CRS	riskstratification by CRS	correctclassification by CRS
#1	yes	21.77	8.40	3.12	1.69	23.14	high risk	yes
#2	yes	20.33	7.02	2.59	1.67	18.95	high risk	yes
#3	yes	10.30	9.82	3.46	1.50	28.73	high risk	yes
#4	yes	8.63	8.10	3.11	2.02	21.17	high risk	yes
#5	yes	3.,47	6.68	2.54	1.74	17.42	low risk	no
#6	yes	1.50	8.96	3.86	1.19	23.83	high risk	yes
#7	yes	29.37	7.73	3.12	1.69	20.08	high risk	yes
#8	no	36.00	7.57	3.83	2.64	14.73	low risk	yes

CRS calculated by (4.592× ΔCt miR-21)+(−3.982× ΔCt miR-126)+(−1.938× ΔCt miR-221); high risk if CRS >18.7; low risk if CRS<18.7;

## Discussion

Looking at ccRCC, only a minority of patients with advanced tumors develop TT, resulting in limited numbers of available ccRCC/TT study collectives. This might be one reason why up to date, molecular and genetic changes causing the development of vena cava extensions are poorly understood and applicable prognostic marker systems in ccRCC/TT are still missing. Recent studies have shown that miR expression profiling represents a useful tool to elucidate the genetic and molecular basis of cancer development and progression including ccRCC [20]. Comparison of current miR expression studies revealed that the observed miR profiles of ccRCC are highly reproducible among different patient cohorts, suggesting a possible application of specific miRs as diagnostic or prognostic biomarkers [13], [18], [21], [22], [23], [24]. Based on these studies, we selected a panel of eight different miRs, which were previously described to be differentially expressed or to be correlated with progression in ccRCC and determined their expression in our ccRCC/TT study collective. Our aim was to generate a basis for the development of new diagnostic and prognostic tools in this important subgroup. As expected, we confirmed differential expression of miR-21, miR-210, miR-141, miR-200c and miR-126 in ccRCC. Using the expression data of five miRs (miR-21, miR-143, miR-200c, miR-210 and miR-126), we were able to separate ccRCC from normal kidney tissue with an accuracy of 100%, indicating that our panel of miRs is related to ccRCC development. Those results are in line with the observed tumorigenic function of the selected miRs and with recent studies successfully discriminating RCC from normal tissue by the use of specific miR profiles [18], [19], [25].

Next, we presented results focusing on the establishment of a discriminative miR profile to distinguish ccRCC/TT from ccRCC/woTT. To date, no attempt has been made to classify ccRCC/TT from ccRCC/woTT samples using miRs. We observed that three miRs of our miR panel (miR-21, miR-126, miR-221) were significantly regulated in ccRCC/TT. Moreover, using the expression level of three miRs (miR-21, miR-221 and let-7b) we successfully identified ccRCC/TT cases with an accuracy of 94%. From these results, we concluded that among the eight selected miRs miR-21, miR-126, miR-221 and let7b might be critically involved in the development of ccRCC with TT. Several studies already gave evidence that all four dysregulated miRs are involved in biological processes controlling malignant transformation and progression of tumor cells by the identification or prediction of various target mRNAs and pathways controlled by these miRs [13], [20], [26], [27], [28], [29]. Even if our study is limited by the lack of functional and molecular analysis concerning the interaction between miRs and potential target genes, the presented data might provide the basis for further investigations. One common hypothesis is that ccRCC/TT is an intermediate stage between localized ccRCC and a metastasized ccRCC. This hypothesis is supported by the observation that these miRs have been proposed previously to be involved in formation of ccRCC metastasis [13], [20], [30], [31], [32]. To understand how dysregulation of these miRs might mediate venous invasion, aggressiveness or both in renal cancer cells via posttranscriptional regulation of potential target genes, it will be necessary to perform functional studies using *in vitro* and *in vivo* models in the future.

An aggressive surgical approach is the only hope for curing ccRCC patients with any level of TT. Many reports demonstrated that a subgroup of patients with TT can achieve long term survival after aggressive surgical treatment, suggesting that TT invasion in the venous system is not necessarily associated with worse prognosis and aggressiveness of the tumor at the time of surgery. These results match our observation as our ccRCC/TT study collective contains a patient subgroup characterized by low risk for progressive disease. Nonetheless, patients with ccRCC/TT generally characterized as high risk patients having a relative poor prognosis. We observed that a significant proportion of ccRCC/TT patients developed early cancer recurrence while others could be characterized by a long term disease free survival indicating the importance of an additional risk stratification model to accurately select patients who may benefit from early and intensified adjuvant therapy. Therefore, one of the critical issues in treatment of ccRCC/TT patients is the development of an accurate predicting model system in these patients. The impact of a number of prognostic nomograms typically including clinico-pathological variables like TNM staging, tumor grade, performance status and serum blood markers (hemoglobin, calcium, lactate dehydrogenase, platelets, neutrophiles and c-reactive protein) have been suggested for risk stratification in ccRCC/TT, but so far none of these models achieve the status of an independent, reliable and applicable predictor system in ccRCC/TT [9]. To improve the accuracy of a predictive model system, it might be helpful to establish molecular biomarkers in addition to standard factors already applied. Systems using molecular biomarkers (e.g. miRs) or a combination of molecular biomarkers with standard clinic-pathological factors (e.g. IMP-3 and tumor staging), have been shown to accurately predict progression and survival in ccRCC tumors [33], [34]. Nevertheless, up to now such predictive molecular markers were not analyzed or identified in ccRCC/TT patients. Here we show that a CRS calculated by the expression of three miRs (miR-21, miR-126 and miR-221) has the potential to classify ccRCC/TT patients that are at high or low risk to develop aggressive disease. Surprisingly, even if we could show that some standard clinic-pathological factors (lymph node metastasis, distant metastasis and tumor grade) have predictive potential in our study collective, the developed predictive model did not use any of these factors implicating that the used CRS is able to classify patients at high risk with pinpoint precision. The predictive power of the determined risk score model is further supported by the observation that 36 ccRCC/woTT are correctly classified as low risk cases by the CRS. This observation is in conclusion with our previous study demonstrating that the expression miR-21 and miR-126 is associated with CSS in ccRCC/woTT [13]. Although currently unable to validate the potential of the CRS in a large independent control ccRCC/TT study group, the predictive power of the CRS for ccRCC/TT might be further confirmed by the correct risk stratification of seven out of eight independent RCC/TT patients. The potential involvement of miR-21, miR-126 and miR-221 in regulation of progression and aggressiveness is also supported by recent studies. Thus, miR-221 down-regulation was recently described as a prognostic marker in high risk prostate cancer controlling the interferon signal pathway in cancer cells and was found to be under-expressed in metastatic ccRCC cases [28], [30]. Dysregulation of miR-21 and miR-126 was demonstrated in various cancer types including ccRCC showing involvement of these miRs in important tumorigenic pathways controlling proliferation, angiogenesis, differentiation and migration [21], [26], [31], [35], [36]. Moreover, we have recently shown that a CRS of miR-21 and miR-126 is correlated with survival in ccRCC [13]. Thus, the determined CRS based on the expression of the three onco-miRs demonstrates a possible molecular model to classify ccRCC/TT samples into risk groups for the first time. Since currently no other predictive molecular marker system exists which can identify ccRCC/TT cases by their risk for its outcome, the generation of a robust and specific prediction model is urgently needed. Although the developed CRS model provides high significance and accuracy for predicting CSS in ccRCC/TT, the power of our conclusions is limited by low number of cases, by the lack of confirmation in a large independent validation cohort and by the retrospective nature of the current study. Therefore, it will be necessary to test the effectiveness and reliability in further studies with enlarged validation cohorts to confirm the potential of the determined risk score as a predictive biomarker and possible molecular assay in clinical setting.

In summary, we successfully characterized ccRCC/TT by a distinct miR profile generated by the expression of eight selected oncogenic miRs. The used miR profile is able to accurately classify ccRCC/TT cases. Differential expression of miR-21, miR-221 and let-7b precisely separated ccRCC/TT cases from ccRCC/woTT indicating their possible function in the development of ccRCC/TT. Moreover, a CRS calculated by the expression of three onco-miRs, miR-21, miR-126, and miR-221 was generated, which accurately predicts CSS in the ccRCC/TT collective and in a small independent RCC/TT patient group. Nevertheless, the power of our conclusion may be limited by the relatively small number of ccRCC/TT cases and has to be validated in large ccRCC/TT cohorts. After further evaluation of the reliability and effectiveness of the developed prediction model, we suppose this prognostic molecular marker system will be able to carefully select RCC/TT patients to whom an adjuvant systemic therapy may be advisable.

## Supporting Information

Figure S1 **Expression of miR-21, miR-210, miR-200c, miR-141, miR-126, let-7b miR-221, and miR-143 in ccRCC.** Relative expression (ΔC_t_ levels) of indicated miRs were measured by qRT-PCR and normalized against RNU6B and are shown as Box and Whisker-Plot. Expression of different miRs in ccRCC (RCC, dark grey plots; n = 74) are compared with non-cancerous renal tissue (ctrl.; light grey plots, n = 32). * P<0.05; ** P<0.01; *** P<0.001, Student's t-test.
(TIF)

Figure S2 **Kaplan Meier survival analysis for CSS of renal cell cancer with tumor thrombus patients (n = 37) median follow up of the study group was 41.4 months.** The 5-yr CSS estimate was 43%. CRD: cancer related death; FU: follow up.
(TIF)
